# Therapeutic protein PAK restrains the progression of triple negative breast cancer through degrading SREBP-1 mRNA

**DOI:** 10.1186/s13058-023-01749-7

**Published:** 2023-12-11

**Authors:** Pan Hu, Peiyi Zhou, Tieyun Sun, Dingkang Liu, Jun Yin, Lubin Liu

**Affiliations:** 1 Department of Obstetrics and Gynecology, Women and Children’s Hospital of Chongqing Medical University, No.120 Longshan Road, Yubei District, Chongqing, 401147 China; 2 Jiangsu Key Laboratory of Druggability of Biopharmaceuticals and State Key Laboratory of Natural Medicines, School of Life Science and Technology, China Pharmaceutical University, Nanjing, 210009 China

**Keywords:** Therapeutic protein, Lipogenesis, Fatty acid, SREBP-1, Triple negative breast cancer

## Abstract

Triple-negative breast cancer (TNBC) represents the most challenging subtype of breast cancer. Studies have implicated an upregulation of lipid synthesis pathways in the initiation and progression of TNBC. Targeting lipid synthesis pathways may be a promising therapeutic strategy for TNBC. Our previous study developed a therapeutic protein PAK with passive targeting and inhibiting tumor proliferation. In this study, we further substantiate the efficacy of PAK in TNBC. Transcriptome sequencing analysis revealed PAK-mediated downregulation of genes involved in fatty acid synthesis, including key genes like SREBP-1, FASN, and SCD1. RNA immunoprecipitation experiments demonstrated a significant binding affinity of PAK to SREBP-1 mRNA, facilitating its degradation process. Both in vitro and in vivo models, PAK hampered TNBC progression by downregulating lipid synthesis pathways. In conclusion, this study emphasizes that PAK inhibits the progression of TNBC by binding to and degrading SREBP-1 mRNA, revealing a new strategy for regulating lipid synthesis in the intervention of TNBC and its therapeutic significance.

**Supplementary Information:**

The online version contains supplementary material available at 10.1186/s13058-023-01749-7.

## Introduction

Triple-negative breast cancer (TNBC) is the most challenging subtype of malignant breast tumors, representing approximately 16% of all instances [1]. The defining characteristic of TNBC is the absence of expression of estrogen receptors (ER), progesterone receptors (PR), and human epidermal growth factor receptor 2 (HER2), which renders it unresponsive to existing targeted therapies. Recent research reports have indicated that regulation of lipid metabolism is a key component of metabolic reprogramming in TNBC [2–4]. TNBC cells exhibit excessive activation of de novo fatty acid synthesis pathways [5, 6], leading to lipid accumulation during cell proliferation. Increased fatty acid synthesis contributes to the generation of structural lipids required for cell membrane formation, such as cholesterol and phosphatidylglycerol [7]. Triglycerides are stored in droplet-like structures and serve as energy reservoirs. Additionally, specific types of lipids act as second messengers (e.g., diacylglycerol and phospholipids), facilitating signal transduction [8, 9]. Therefore, targeting lipid synthesis pathways represents a promising strategy for treating TNBC.

Sterol regulatory element-binding protein 1 (SREBP-1) is a key transcription factor involved in lipid biosynthesis, regulating lipid synthesis and uptake, playing a central role in both physiological and pathological conditions [10]. SREBP-1 and various enzymes involved in fatty acid synthesis under its control, such as Fatty Acid Synthase (FASN), Acetyl-CoA Carboxylase α (ACACA), and Stearoyl-CoA Desaturase 1 (SCD1), are significantly upregulated [11–14]. Studies have shown that blocking the SREBP-1 pathway by inhibiting SREBP Cleavage Activating Protein (SCAP) can notably inhibit the progression of diethylnitrosamine-induced hepatocellular carcinoma (HCC) [15]. The novel SREBP-1 inhibitor Fatostatin inhibits the growth, colony formation, invasion, and migration of LNCaP and C4-2B prostate cancer cells, inducing cell cycle arrest and apoptosis by activating caspase-3/7 and cleaving Poly ADP-Ribose Polymerase (PARP) [16]. In glioblastoma multiforme (GBM), EGFRvIII-dependent PI3K/SREBP-1 pathway activation drives de novo fatty acid synthesis, promoting tumor growth and survival [17]. Conversely, liver X receptor (LXR) agonists induce GBM cell death by inhibiting the EGFR/AKT/SREBP-1 pathway [18]. The above evidence indicates that pharmacological inhibition of SREBP-1 may be a potential approach to suppress TNBC progression.

Our group had previously developed a novel therapeutic protein called PAK, which exhibited prolonged, tumor-targeted release and demonstrated effective antitumor effects in both in vitro and in vivo models [19, 20]. This study further confirmed the efficacy of PAK in TNBC. Remarkably, PAK exhibited significantly higher inhibitory activity against TNBC cells compared to other breast cancer cell lines. Transcriptome sequencing analysis revealed that PAK downregulated the transcription of genes involved in fatty acid synthesis, including key genes SREBP1, FASN, SCD1, and ACACA. This suggests that PAK may suppress tumor progression in TNBC cells by downregulating de novo fatty acid synthesis. Although its pro-apoptotic effect is well defined [19, 21], the mechanism by which PAK regulates fatty acid synthesis remains unclear. Based on these findings, we hypothesized that PAK inhibits the cell lipid synthesis pathway by downregulating SREBP-1 levels, thereby suppressing TNBC proliferation.

In this study, we first determined the impact of PAK on the viability, invasion, and migration of breast cancer cells. Subsequently, based on the results of transcriptome sequencing analysis, we screened and validated key target genes affected by PAK and further confirm their role in PAK anti-tumor activity at both cellular and animal levels. Ultimately, we aim to elucidate how PAK inhibits the progression of triple-negative breast cancer by regulating the de novo synthesis pathway of fatty acids.

## Materials and methods

### Cell lines

MCF7, SKBR-3, MDA-MB-468 and MDA-MB-231 were purchased from ATCC and maintained in RPMI 1640 medium (10% FBS). Cells were incubated at 37 °C with 5% CO_2_. All cell lines have been authenticated using short tandem repeat profiling within the last three years. All experiments were performed with mycoplasma-free cells.

### Plasmids and transfection

Plasmid of pcDNA3.1(+)-SREBP-1 was synthesized by GenScript (Nanjing, China). Transfection was performed using Lipo3000 DNA Transfection Reagent (Thermo Scientific, Massachusetts, USA) and followed the manufacturer’s instructions.

### Antibodies and reagents

Antibodies used in the research were Anti-SCD1 from Cell Signaling Technology; Anti-FASN/β-actin/vimentin/N-cadherin and Anti-E-cadherin from Proteintech; Anti-SREBP-1 from BD Pharmingen. Palmitic Acid (16:0) (PA), Oleic acid (OA) were purchased from Solarbio life sciences. Cell Counting Kit-8, Actinomycin D, Nile Red, and crystal violet were purchased from MedChemExpress. Matrigel matrix was purchased from Corning.

### Western blot analysis

Cells were washed and then scraped into RIPA buffer (Beyotime, Nantong, China). Protein extracts controlled by β-Actin were separated by 10% SDS-PAGE. The wet transfer method was used to transfer the protein from gels to PVDF membranes (Beyotime, Nantong, China). After blocking in TBST buffer containing 5% nonfat milk, membranes were incubated with corresponding primary antibodies all night and then probed with a secondary antibody coupled to horseradish peroxidase for 1 h. The PVDF membranes were detected by an ECL kit (Merck Millipore). Densitometry was quantified by using ImageJ Software.

### mRNA sequencing by Illumina HiSeq and KEGG enrichment analysis

For each sample, total RNA was extracted with TRIzol reagent (Invitrogen). Total RNA per sample was analyzed qualitatively and quantitatively by Agilent 2100 Bioanalyzer (Agilent Technologies, USA), NanoDrop (Thermo Scientific, Massachusetts, USA), and 1% agarose gel. Sequencing libraries were prepared using 1 μg of total RNA with a RIN value of 7 or higher. Next-generation sequencing library preparation was performed under the manufacturer's protocol (NEBNext® Ultra™ RNA Library Prep Kit for Illumina®). Libraries with different exponents were then multiplexed and imported onto the Illumina HiSeq instrument (Illumina, San Diego, CA, USA) following the instructions provided. Sequencing was performed using 2 × 150 bp paired ends (PE); analysis of images and bases was performed on HiSeq Control Software (HCS) + OLB + GAPipeline-1.6 (Illumina). Sequences were processed and analyzed by GENEWIZ. KEGG (Kyoto Encyclopedia of Genes and Genomes) is a database that integrates genomic, chemical, and phylogenetic functional information. (http://en.wikipedia.org/wiki/KEGG). We used it to compare genomic profiles and look for significantly differentially expressed genes. The raw RNA-seq data was uploaded to NCBI SRA database (SRA accession number: SRR13399489).

### RNA extraction and qPCR analysis

Total RNA was obtained by using EasyPure® RNA Kit (TransGen Biotech) for monolayer cell RNA extraction. 800 ng RNA was subjected to reverse transcription to cDNA using HiScript SuperMix (Vazyme, Nanjing, China) and amplified through qPCR using qPCR Master Mix (Vazyme, Nanjing, China). The primers used were: SREBP1 forward: 5′-CGC TCC TCC ATC AAT GAC A-3′, reverse: 5′-TGC GCA AGA CAG CAG ATT TA-3′; ACLY forward: 5′-GAA GGG AGT GAC CAT CAT CG-3’, reverse: 5’-TTA AAG CAC CCA GGC TTG AT-3′; FASN forward: 5′-GTT CAC GGA CAT GGA GCAC-3′, reverse: 5′-GTG GCT CTT GAT GAT CAG GTC-3′; SCD1 forward: 5′-TGC GAT ATG CTG TGG TGCT-3′, reverse: 5′-GAT GTG CCA GCG GTA CTCA-3′; ACSS2 forward: 5′-TGA AAC CCG GTT CTG CTA CT-3′, reverse: 5′-TCT CAA AGC GTT CGT GGT TC-3′. The amplification procedures were as follows: 95 °C for 180 s, then denatured at 95 °C for 10 s, annealing at 62 °C for 20 s with 40 cycles.

### Cell cycle and apoptosis analysis

MDA-MB-231 cells were incubated with PBS or PAK and then fixed by paraformaldehyde and stained with nuclear dye PI (Beyotime, Nantong, China), and analyzed by flow cytometry for cell cycle distribution determination. The apoptotic cell was assessed using AV-FITC/PI Apoptosis Detection Kit (Vazyme, Nanjing, China). The results were analyzed by using FlowJo_V10 (BD, USA).

### Wound healing assay

The individual wells were wounded by scratching with a 200 μL pipette tip. PBS was used to wash and remove the floating cells. The cells were photographed at 0 h, 24 h, and 48 h, and the area occupied by migrated cells was measured by using ImageJ Software.

### Cell invasion and migration assays

The assay was determined using a 24-well plate consisting of 8 μm membrane filters (Corning Inc., USA). Besides, Matrigel was used for filter membrane coverage for the invasion assay. 1 × 10^5^ cells were added into the upper chamber and 650 μL medium was added to the lower chamber. After incubation for 24 h, cells on the lower surface of the membrane were fixed by paraformaldehyde and stained with crystal violet.

### Mouse xenograft experiments

All animal care and experimental protocols complied with the Laboratory Animal Management Regulations in China and the ethical committee at China Pharmaceutical University (license No. SCXK (su) 2016-0003). Animal studies are reported in compliance with the ARRIVE guidelines. 5-week-old female BALB/c nu/nu mice were purchased from Vital River (Beijing, China). The tumor-bearing model was established by the injection of MDA-MB-231 cells (3 × 10^6^ cells/mouse) in the flank of mice. When tumor size was about 100 mm^3^, 25 mice were randomly divided into 5 groups and treated with PBS, DOX (4 mg/kg, every week), and PAK (0.25, 0.5, 1 μmol/kg, every 3 days) by intraperitoneal injection based on the pre-experiment data and literature reported [19, 20]. During the treatment, tumor volumes were calculated according to a simplified formula: 0.5 × length × width^2^. At the end of treatment, mice blood was sampled to assay heart and liver enzymes and hematological parameters, while tumors and tissues were isolated and stained for immunohistochemistry, TUNEL, and H&E staining by Servicebio (Nanjing, China).

### Statistics

Data are expressed as the mean ± SD. Statistical analyses were conducted by using GraphPad Prism 7 (GraphPad Software). Significant difference analysis was determined using Student's t-test (two group) or one-way ANOVA followed with Bonferroni’s multiple comparisons test (more than two groups). ***P < 0.001, **P < 0.01, and *P < 0.05 was considered significant.

## Results

### PAK inhibits the progression of TNBC cells in vitro

**Fig. 1:**
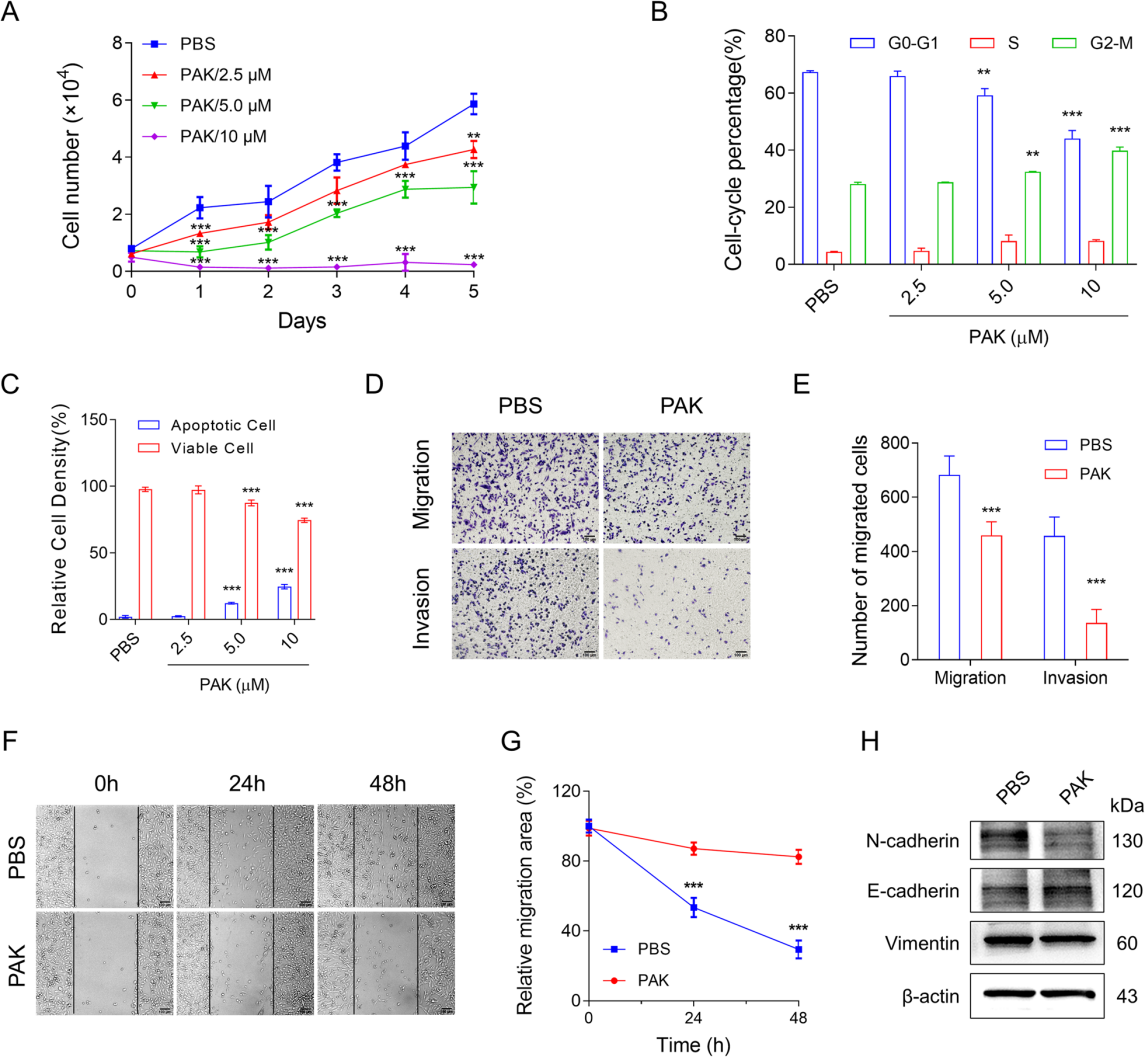
PAK inhibits the proliferation, invasion, and migration of TNBC cells in vitro. **A** Under the treatment of PAK, cell proliferation of MDA-MB-231 was analyzed using CCK8 assay. **B** After 24 h treatment, the effect of PAK on MDA-MB-231 cell-cycle distribution was measured by flow cytometry. **C** Relative cell densities of viable cells and apoptotic cells (Annexin V-positive) were compared to PBS-treated group after 24 h of treatment. **D–E** For the Transwell assay, PAK (5 µM) was applied to MDA-MB-231 cells for 24 h. Cell invasion and migration were photographed and quantified, respectively. Scale bar: 100 μm. **F–G** For the wound healing experiment, PAK (5 µM) was applied to MDA-MB-231 cells for 48 h. Scale bar: 100 μm. **H** Under the treatment of PAK (5 µM), EMT in MDA-MB-231 cells are detected by Western Blot, including N-cadherin, E-cadherin, and Vimentin. Data shown are mean ± SD (n = 5). **P < 0.01; ***P < 0.001; *vs* PBS-treated group

To investigate the antitumor effect of PAK on human breast cancer cells, MDA-MB-231, MDA-MB-468, SKBR-3, and MCF7 cells were treated with PAK at a series of concentrations. Firstly, we investigated the cytotoxic effects of PAK on the aforementioned cell lines using the CCK8 assay. Surprisingly, PAK exhibited a significantly higher inhibitory effect on MDA-MB-231 and MDA-MB-468 cells compared to SKBR3 and MCF7 cells (Additional file 1: Figure S1). The results of further colony formation assays were consistent with the findings from CCK8 assay (Additional file 1: Figure S2). Given the relatively better therapeutic efficacy of PAK in two types of TNBC cell lines, we employed MDA-MB-231 as a representative TNBC cell line for further investigation. Figure 1A showed that PAK exhibited dose (0–10 µM) and time (0–5 days) dependent growth inhibition in MDA-MB-231 cells. Cell cycle distribution analysis was subsequently performed. As depicted in Fig. 1B and Additional file 1: Figure S3, PAK demonstrated a dose-dependent accumulation in the G2-M phase of the cell cycle in MDA-MB-231 cells. Furthermore, the activation of apoptosis in MDA-MB-231 cells after PAK incubation was measured by flow cytometer. Significant apoptosis was observed when the dosage was increased to 5 μM (Fig. 1C, Additional file 1: Figure S4). Additionally, the number of necrotic cells also increased with higher doses of PAK. The above results showed that PAK inhibits the proliferation of TNBC cells in vitro.

Progressive and metastatic tumor cells possess the capacity to infiltrate and migrate into surrounding tissues. Epithelial-mesenchymal transition (EMT) is considered a pivotal step in cancer metastasis [22]. In light of this, we investigated the impact of PAK on the invasive and migratory capabilities of MDA-MB-231 cells. Following a 24-h treatment, PAK (5 µM) significantly attenuated invasion and migration abilities in comparison to the untreated group (Fig. 1D, E). The wound healing assay further confirmed the suppressive role of PAK in cellular migration (Fig. 1F,G). Immunoblot analysis of EMT-associated markers, as illustrated in Fig. 1H, revealed that PAK treatment led to an increase in E-cadherin expression while reducing N-cadherin and Vimentin levels. Overall, these findings collectively indicate that PAK inhibits the proliferation, invasion, and migration of TNBC cells.

### PAK inhibits the progression of TNBC in vivo

To further assess the in vivo anti-tumor efficacy of PAK against TNBC, we established a xenograft mouse model using MDA-MB-231 cells. Firstly, we validated the ability of PAK to effectively target TNBC tumor cells. As shown in Additional file 1: Figure S5A, Cy5-labeled PAK protein demonstrated excellent tumor targeting capability at 3.5 h and enhanced fluorescence intensity at 4 h. Additional file 1: Figure S5B-C shows the ex vivo imaging results at the 4-h time point, including heart, liver, spleen, lung, kidney, and tumor tissues. Consistent with previous studies, the accumulation of PAK was also observed in the kidney, one of the major metabolic organs. Safety data from our previous study showed no toxicity of PAK in kidney [19, 20]. These results indicated that PAK can accumulate in tumor tissues, and the excess drug is primarily cleared through the kidney.

**Fig. 2:**
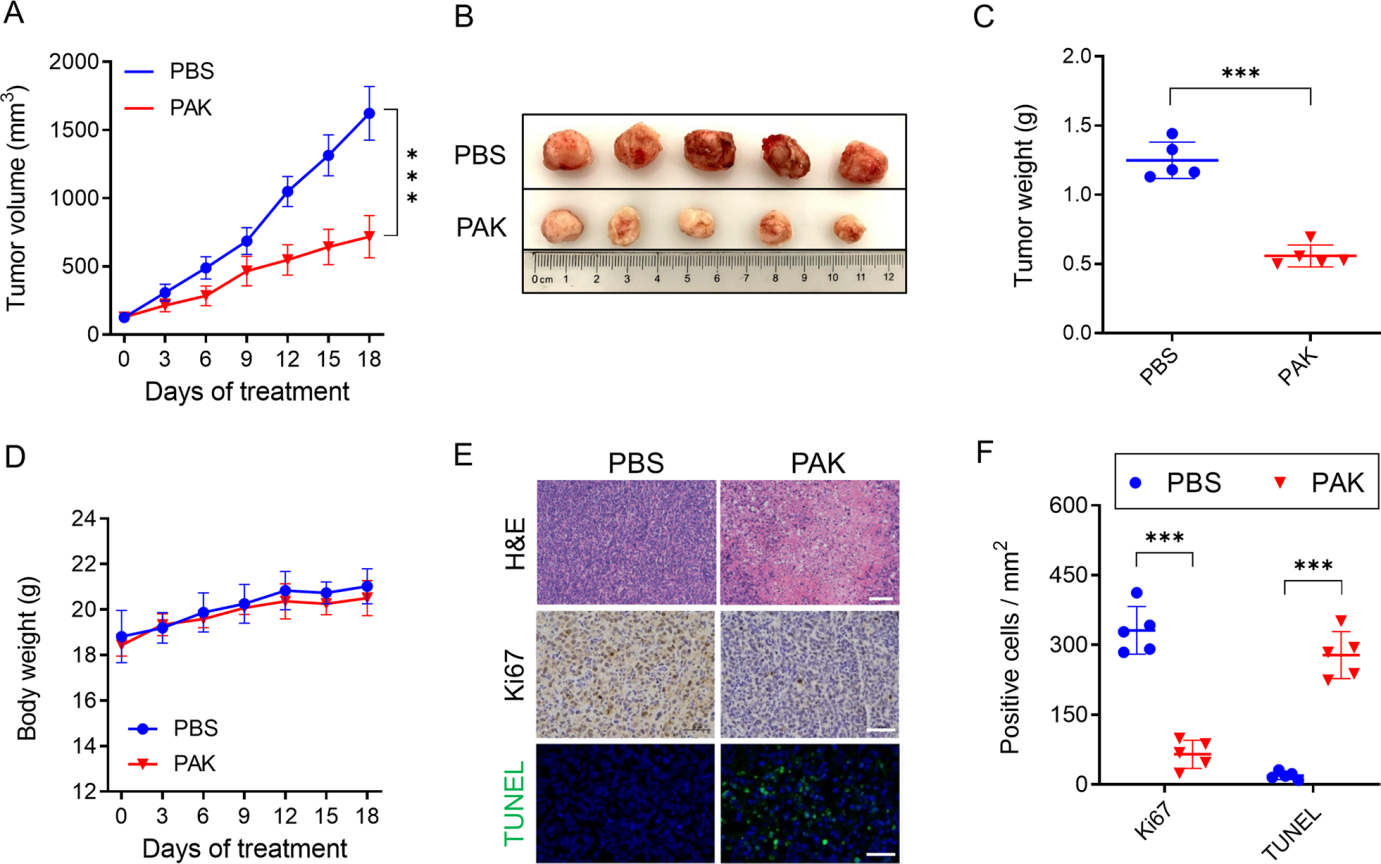
PAK exhibits antitumor effects against TNBC in vivo. **A** Under the treatment of PAK (1 μmol/kg, every 3 days, *i.v* injection), tumor volumes were measured every three days. **B**, **C** Tumor tissues were collected on day 18 after treatment, and tumor images and weights were recorded. **D** Body weight of each mouse was measured every three days. **E** Immunohistochemical analysis was conducted to measure the Ki67 level in tumor tissue. Frozen tumor tissue sections were conducted with TUNEL apoptosis assay. Scale bars, 50 μm. **F** Quantitative analysis of Ki67 level and TUNEL staining. Data are mean ± SD (n = 5). *P < 0.05; **P < 0.01; ***P < 0.001; n.s., no significant; *vs* PBS-treated group

Next, the MDA-MB-231 xenograft mice were divided into two groups: PBS-treated group, and PAK-treated group (1 μmol/kg). After 18 days of treatment, the tumor volumes of PAK-treated groups exhibited significant suppression in comparison to the PBS-treated group (Fig. 2A,B). Tumor weights paralleled the tumor volume trends (Fig. 2C). Throughout the treatment period, the body weights of the both groups displayed slight increments (Fig. 2D). Meanwhile, the results of H&E staining showed that PAK demonstrated significantly inhibitory effect of the MDA-MB-231 tumor (Fig. 2E,F). Moreover, compare to the PBS-treated group, PAK-treated group displayed a substantial increase in TUNEL positive cells within the tumor tissue (Fig. 2E,F), signifying the induction of tumor apoptosis by PAK. Furthermore, the PAK-treated group exhibited notably lower Ki67 antigen expression levels within tumor tissues compared to the PBS-treated (Fig. 2E,F). These findings collectively suggested that PAK exhibits antitumor effect in the MDA-MB-231 xenograft model.

### PAK inhibits fatty acid synthesis through degrading SREBP1 mRNA

**Fig. 3:**
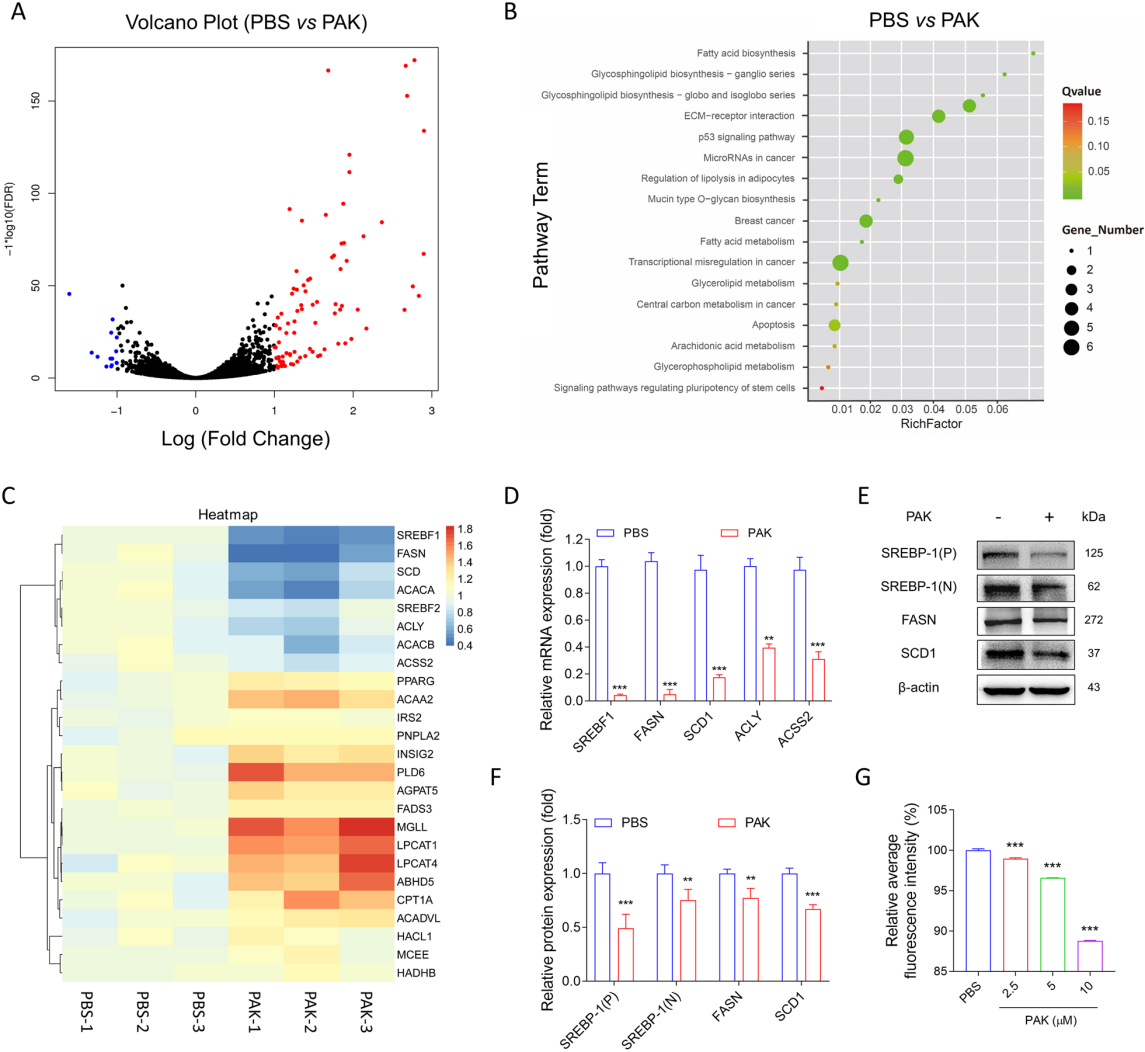
Transcriptomic analysis after PAK treatment in MDA-MB-231 cells. Cells were obtained for RNA-seq analysis after treatment with PBS or PAK for 24 h. **A** Analysis software generated the volcano plot of differentially expressed genes, where the red dots are up-regulated and the blue dots are down-regulated. **B** KEGG pathways highly associated with changes resulting from PAK treatment. **C** Gene expression calculation uses HiSeq Analysis Software (v0.9), which uses FPKM [36] method to calculate gene expression. **D** Relative mRNA expression of SREBP1 target genes from MDA-MB-231 cells treated with PBS or PAK for 24 h was measured. Data were normalized with β-actin. **E, F** Protein levels were measured by Western blotting in MDA-MB-231 cells treated with PBS or PAK for 24 h. **G** Nile Red fluorescence intensity in MDA-MB-231 cells treated with PBS or PAK for 24 h was measured. Data shown are mean ± SD (n = 3). *P < 0.05; **P < 0.01; ***P < 0.001; versus PBS-treated group

To elucidate the pharmacological mechanism of PAK, we conducted RNA-seq analysis on MDA-MB-231 cells treated with PBS or PAK, and the principal component analysis of RNA-Seq were shown in Additional file 1: Figure S6. RNA-seq analysis was filtered according to the significance criteria for differential expression (fold change > 2 and q-value < 0.05). The significant differential expression of genes in both upregulated and downregulated directions was then quantified (Fig. 3A and Additional file 1: Figure S7). Differential gene KEGG enrichment results found that PAK negatively regulated fatty acid synthesis genes FASN, SCD1, and ACLY (Fig. 3B,C), hence we further focused on the pathways of lipid metabolism. Genes associated with lipid metabolism were systematically screened, with emphasis on those displaying significant expression alterations. Upon comparative expression analysis, the attenuation of SREBP1 and FASN emerged as notably pronounced (Additional file 1: Figure S8). Consequently, we hypothesized that PAK inhibits tumorigenesis in MDA-MB-231 cells by suppressing fatty acid synthesis pathways. Employing qPCR and Western blot assays, we observed a substantial reduction in the mRNA levels (Fig. 3D) and protein levels (Fig. 3E,F) of FASN, SREBP-1, and SCD1 due to PAK treatment. Moreover, PAK induced a decrease in both the precursor (P) and cleaved nuclear forms (N) of SREBP-1 protein expression (Fig. 3F). Subsequently, employing the lipophilic dye Nile Red for lipid visualization and quantification, intracellular lipid levels were assessed via Nile Red staining. The outcomes demonstrated a dose-dependent reduction in intracellular lipid content by PAK (Fig. 3G, Additional file 1: Figure S9). However, the precise mechanism by which PAK downregulates SREBP-1-mediated lipid synthesis remains elusive.

**Fig. 4:**
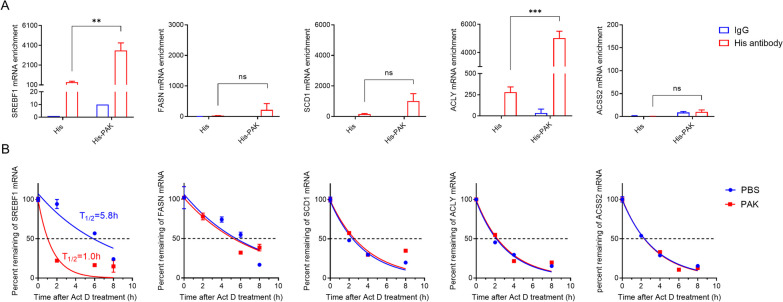
Interaction of PAK with SREBP1 pathway mRNA. **A** RNA immunoprecipitation was used to detect the binding of protein and mRNA. **B** Decay of mRNA was monitored in MDA-MB-231 cells treated with Actinomycin D and PAK. The decrease in mRNA levels was determined by qPCR analysis for each time point. Data were fitted by Nonlinear regression. Data shown are mean ± SD (n = 5). *P < 0.05; **P < 0.01; ***P < 0.001; n.s., no significant

Given the downregulation of mRNA levels of lipid synthesis pathway, we postulated a potential direct interaction between PAK and mRNA of SREBP1 signal mRNA. To investigate this, an RNA immunoprecipitation (RIP) assay was employed to assess the binding effect between PAK and mRNA. Remarkably, PAK exhibited a significant binding affinity towards SREBP1 and ACLY mRNA, while no binding effect was observed with FASN, SCD1, and ACSS2 (Fig. 4A). Subsequently, RNA stability assays were conducted to ascertain whether PAK could influence the stability of these mRNAs. Results revealed that PAK promoted the degradation of SREBP1 mRNA while exhibiting no impact on the stability of FASN, SCD1, ACLY, and ACSS2 mRNA (Fig. 4B). Collectively, these findings elucidate that within MDA-MB-231 cells, PAK binds to and facilitates the degradation of SREBP1 mRNA, consequently contributing to the downregulation of its protein level and subsequent attenuation of downstream fatty acid synthases.

### The antitumor effect of PAK can be reversed by overexpression of SREBP-1

**Fig. 5:**
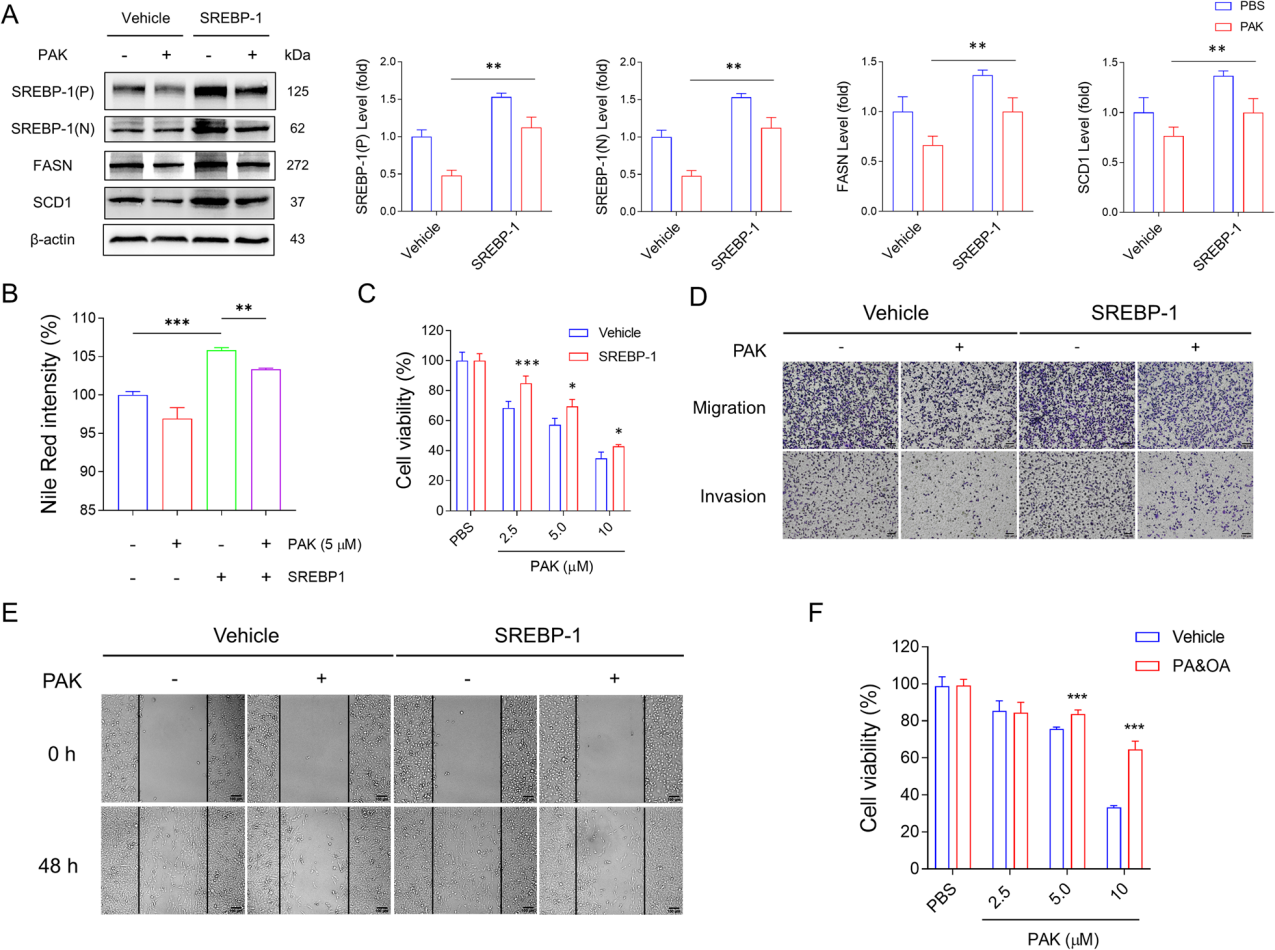
Antitumor effects of PAK can be reversed by SREBP-1 overexpression. After overexpression of SREBP-1 for 24 h, MDA-MB-231 cells were treated with PBS or PAK. **A** Western blot detection of related genes after overexpression of SREBP-1. **B** Nile Red fluorescence intensity distribution in MDA-MB-231 cells treated with PAK or/and OE-SREBP-1 was measured. **C** CCK8 assay was applied to measure the relative cell viability of each group. **D** Transwell assay was conducted. The photographs show images of cells on the underlayer of the membrane. Scale bar: 100 μm. **E** Wound healing experiment showed the condition of migrating cells. Scale bar: 100 μm. **F** MDA-MB-231 cells were treated with PBS or PAK for 24 h. PA&OA or vehicle was added into the cell culture medium additionally. CCK8 assay was used to determine relative cell viability. Data shown are mean ± SD (n = 5). *P < 0.05; **P < 0.01; ***P < 0.001; n.s., no significant

Given the pivotal role of SREBP-1 as an upstream transcriptional regulator of FASN, SCD1, and ACACA, it is postulated that SREBP-1 could be a crucial target in PAK therapy. To validate this hypothesis, we introduced the plasmid pcDNA3.1(+)-SREBP-1 into MDA-MB-231 cancer cells. Overexpression of SREBP-1 led to elevated levels of SREBP-1, FASN, and SCD1. However, protein levels remained influenced by PAK, akin to control cells (Fig. 5A). Concurrently, exogenous overexpression of SREBP-1 in PAK-treated cells significantly ameliorated lipid levels, restoring them to levels comparable to the control group (Fig. 5B, Additional file 1: Figure S10). This reconstituted SREBP-1 augmentation bolstered cellular vitality, enhancing PAK-induced dose-dependent cytotoxicity (Fig. 5C). Assessment of cell invasion and migration revealed that Srebp-1 overexpression, similar to control, reinstated migratory capacity following PAK treatment (Fig. 5D,E). Subsequently, a rescue attempt was undertaken, involving the supplementation of fatty acids to the culture medium. Compared to the vehicle group, the addition of PA and OA alleviated the impact of PAK treatment (Fig. 5F). This rescue endeavor elucidated the capacity of cells to uptake exogenous fatty acids, counteracting the decline in intracellular lipid levels and preventing cell demise. In aggregate, these findings underscore the regulatory role of PAK on SREBP-1 expression, culminating in intracellular lipid reduction and ultimately synergistically hindering cell proliferation and migration.

### PAK inhibits the progression of TNBC through lipid reduction in vivo

**Fig. 6:**
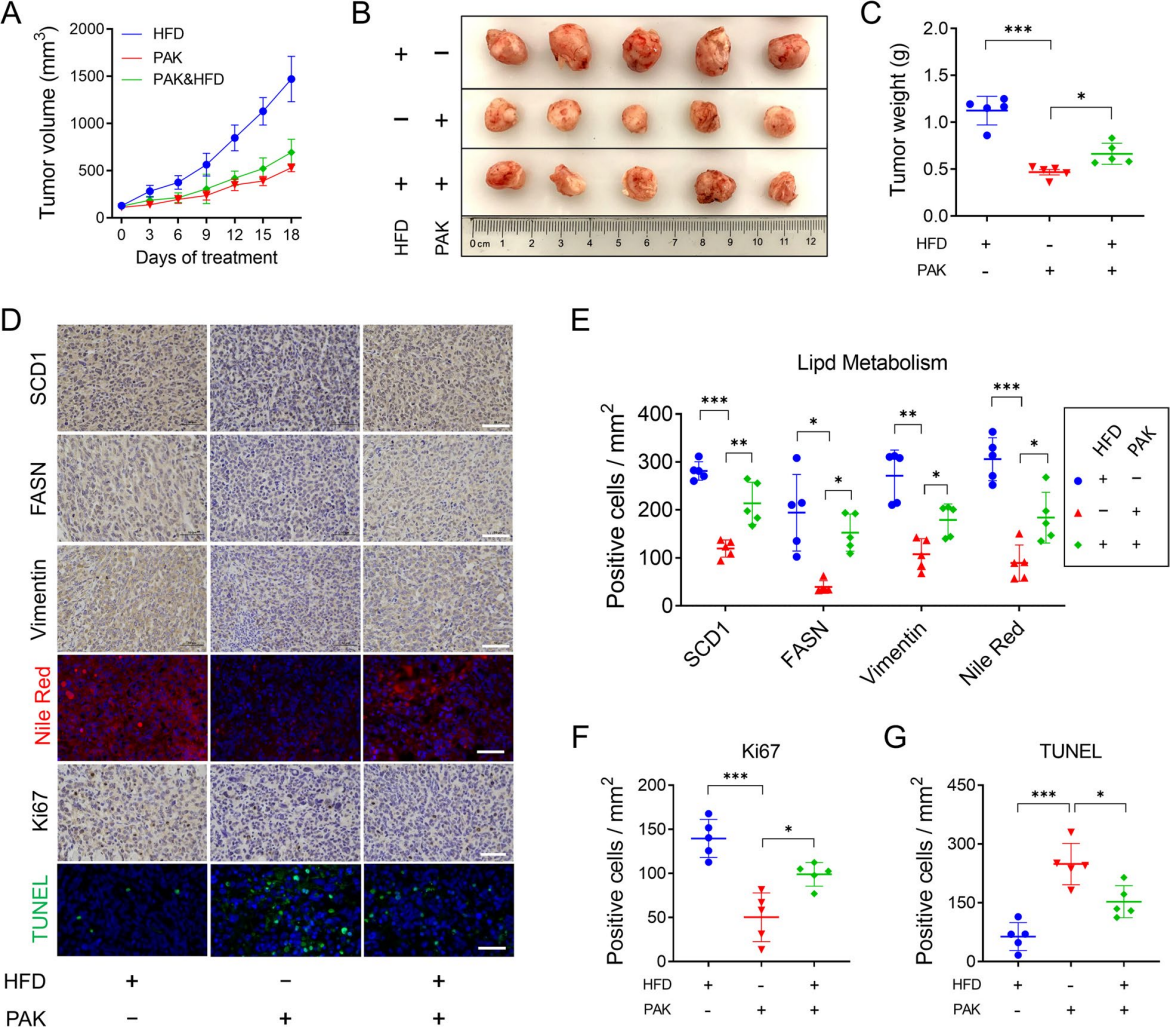
Mechanisms of PAK on lipid level reduction. **A** Under PAK treatment (1 μmol/kg, every 3 days, i.v injection) and high-fat diet (HFD), tumor volumes were measured every three days. **B**, **C** Tumor tissues were collected on day 18 after treatment, and tumor images and weights were recorded. **D** Immunohistochemical analysis was conducted to measure the expressions of FASN, SCD1, Vimentin, and Ki67 in tumor tissue. Frozen tumor tissue sections were conducted with Nile Red and TUNEL staining. Scale bars, 50 μm. **E** Quantitative analysis of SCD1, FASN, Vimentin level and Nile Red staining. **F** Quantitative analysis of Ki67 level. **G** Quantitative analysis of TUNEL staining. Data are mean ± SD (n = 5). *P < 0.05; **P < 0.01; ***P < 0.001; n.s., no significant

To further explore the role of lipid reduction in PAK-induced antitumor, MDA-MB-231 xenografting mice were fed either a normal diet or a high-fat diet (HFD). After 18 days of treatment, the tumor volumes of HFD/PAK-treated groups exhibited fewer suppression in comparison to the PAK-treated group (Fig. 6A,F). Tumor weights closely paralleled the tumor volume trends (Fig. 6C). Immunohistochemical analysis, as depicted in Fig. 6D,E, revealed that PAK downregulated the expression levels of two lipid synthesis enzymes, FASN and SCD1. PAK also downregulated the expression level of Vimentin (an EMT marker) within the tumor tissue. Furthermore, Nile Red staining illustrated that PAK reduced the number of lipid droplets within the tumor tissue. Notably, these results of PAK-induced lipid reduction could be significantly reversed in HFD/PAK-treated group. Moreover, as depicted in Fig. 6D,F, the HFD/PAK-treated group exhibited more Ki67 expression within tumor tissues, compared to the PAK-treated group. Furthermore, compare to the PAK-treated group, the HFD/PAK-treated groups displayed a substantial decrease in apoptotic cells within the tumor tissue (Fig. 6D,G). These findings collectively suggested that PAK inhibits TNBC proliferation and metastasis through the inhibition of lipid synthesis in the MDA-MB-231 xenograft model.

## Discussion

The metastasis of malignant tumors is a primary cause of treatment failure in TNBC. Several discontinuous steps exist within the biological cascade of metastasis, including loss of cell adhesion, increased motility and invasiveness, entry into the systemic circulation, colonization of new tissues, and ultimately survival and proliferation at distant sites [23]. EMT has long been regarded as a pivotal step in cancer metastasis, playing a crucial role in endowing cells with migratory and invasive properties [23]. During this process, cells lose their epithelial characteristics and acquire mesenchymal features, manifested by downregulation of the epithelial marker E-cadherin and upregulation of the mesenchymal markers N-cadherin and Vimentin [24, 25]. In our previous work, the therapeutic protein PAK developed by our team demonstrated effective anti-tumor effects in a melanoma model [19]. In this study, we sought to validate the anti-tumor potential of PAK across various subtypes of breast cancer cell lines. Notably, our investigation revealed particularly promising anti-tumor activity of PAK in TNBC cells. To delve into the underlying mechanisms, we embarked on an in-depth investigation.

Owing to the Warburg effect, tumor cells engage in aerobic glycolysis, yielding glycolytic intermediates that facilitate lipid synthesis and furnish cellular growth processes [26]. Breast cancer and other cancer cell types heavily rely on de novo fatty acid synthesis, essential for membrane phospholipid biosynthesis, signal transduction, and energy generation [27, 28]. The upregulation of these pathways is more pronounced in breast cancer cells than in normal tissues, driving tumorigenesis [29–31]. In this study, we found that PAK, via direct binding to SREBP-1 mRNA, inhibits SREBP-1 expression to regulate lipid metabolism and MDA-MB-231 cell growth and migration. We observed that PAK downregulates the expression of lipogenic genes such as SREBP-1, FASN, and SCD1, leading to a reduction in intracellular fatty acid levels, thereby sustaining the suppression of tumorigenicity. Remarkably, our investigation reveals that MDA-MB-231 cells can uptake exogenous free fatty acids as a compensatory mechanism to counteract the anti-tumorigenic effects of PAK-mediated lipogenic inhibition.

Studies have shown that fatty acids promote cancer proliferation and migration, with the EMT being implicated in this signaling cascade. Cholesterol and phospholipids participate in lipid raft formation, and alterations in membrane rigidity during lipogenesis can modulate cancer cell invasiveness [32]. Moreover, studies have identified that overexpression of SREBP-1 induces a state of EMT, accelerating breast cancer progression [32]. The unsaturated fatty acid synthase SCD1 stimulates cell proliferation through EMT activation, and silencing SCD1 expression can restore the upregulation of EMT phenotypes [33–35]. In this study, we first determined that PAK has a significant ability to inhibit cell invasion and migration. Subsequently, the changes in expression levels of three EMT markers (E-cadherin, N-cadherin, and Vimentin) after PAK incubation indicated that PAK inhibits the EMT process in MDA-MB-231 cells. Overexpression of SREBP-1 can block the inhibitory effect of PAK on cell invasion and migration in MDA-MB-231 cells. Overall, these findings revealed that PAK inhibited the invasive and migratory capabilities of TNBC cells through downregulating the SREBP-1 level.

**Fig. 7:**
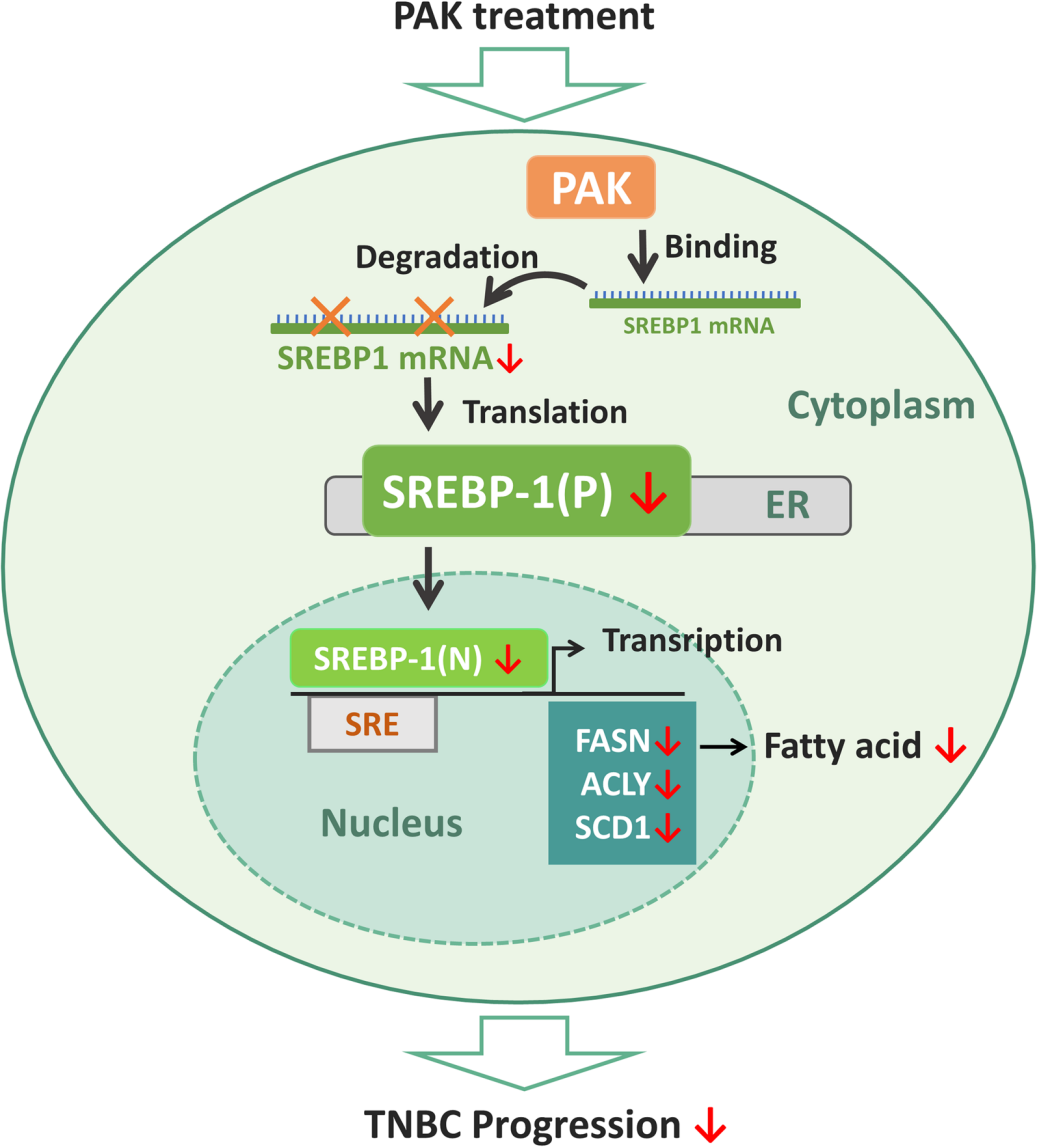
Mechanism Scheme of therapeutic protein PAK on the TNBC

In summary, this study has demonstrated that PAK impedes the progression of TNBC through degrading SREBP-1 mRNA (Fig. 7). We observed that PAK suppresses the fatty acid synthesis in TNBC cell lines and tumor-bearing mouse models. Our study uncovers the functional and mechanistic insights into PAK-mediated regulation of lipid biosynthesis within the TNBC, shedding light on its pivotal role in modulating lipidogenesis and its potential implications for therapeutic interventions.

### Supplementary Information

**Additional file 1.** Supplementary Methods, Figures S1-S10, Images.

## Data Availability

The data that support the findings of this study are available upon reasonable request to the corresponding author.
