# Supplementary material for: Therapeutic protein PAK restrains the progression of triple negative breast cancer through degrading SREBP-1 mRNA
Source: Breast Cancer Res. 2023 Dec 11;25:151. doi: 10.1186/s13058-023-01749-7 (PMC10714641; doi:10.1186/s13058-023-01749-7)
Supplement: Supplementary file 1 — Additional file 1. Supplementary Methods, Figures S1-S10, Images. [file 13058_2023_1749_MOESM1_ESM.docx]

**Supplementary Materials**

**Therapeutic protein PAK restrains the progression of triple negative breast cancer through degrading SREBP-1 mRNA**

*Pan Hu^#1^, Peiyi Zhou^#1^, Tieyun Sun^1^, Dingkang Liu^2^, Jun Yin*^2^, Lubin Liu*^1^*

^1^*Department of Obstetrics and Gynecology, Women and Children’s Hospital of Chongqing Medical University, No.120 Longshan Road, Yubei District, Chongqing, 401147, China*

^2^*Jiangsu Key Laboratory of Druggability of Biopharmaceuticals and State Key Laboratory of Natural Medicines, School of Life Science and Technology, China Pharmaceutical University, Nanjing, 210009, China*

*Corresponding authors:

Lubin Liu, e-mail: liulubin1975@126.com, telephone: +86-18623088100, we designate this one to further communicate with the Editorial and Production offices.

Jun Yin, e-mail: junyin@cpu.edu.cn, telephone: +86-13770339132.

#These authors contributed equally to this work.

1. **Supplement Methods**

**Clone formation assay**

For colony-forming capability analysis, the cells (800 cells/well) were seeded in a 6-well plate, and 1640 medium (10% FBS) containing drugs of all groups were added and incubated according to the corresponding experimental conditions. After being cultured for 10 days, cells were fixed with 4% paraformaldehyde for 15 min. Subsequently, the cells were washed with distilled water 3 times and stained with crystal violet dye solution (Beyotime, Nantong, China) at room temperature for 10 min. Then, the cells were washed with distilled water 3 times and the colonies were counted. The data were expressed as the number of colonies per well.

1. **Supplement Figures**


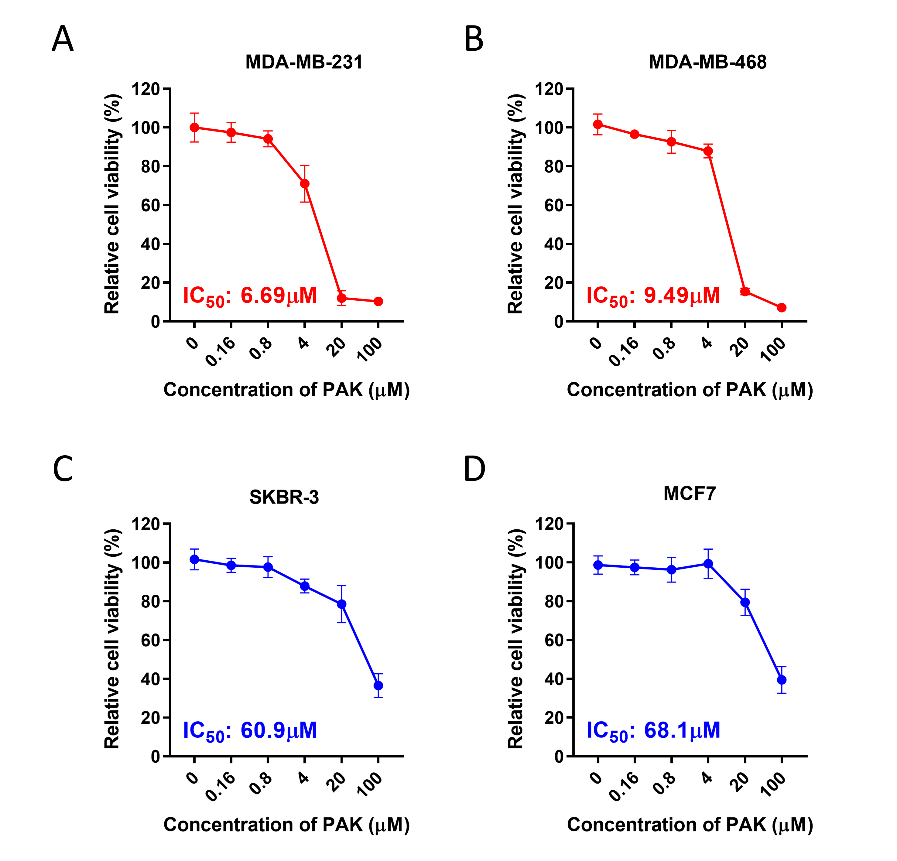


**Figure S1.** PAK was applied at different concentrations in MDA-MB-231 (a), MDA-MB-468 (b), SKBR-3 (c), MCF7 (d) cells for 24 hours. Data are mean ± SD (n = 5).


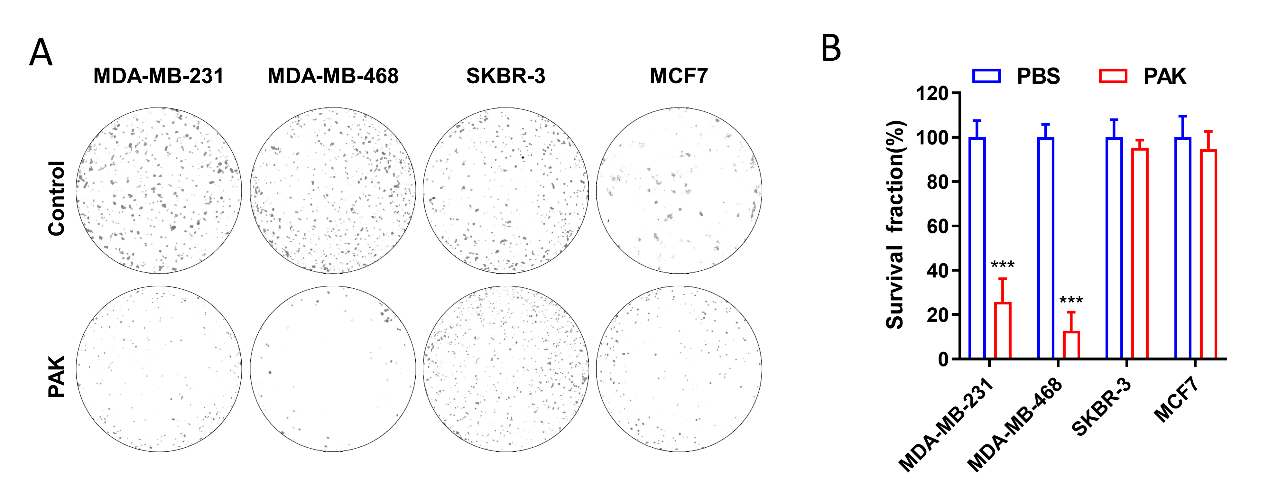


**Figure S2.** PAK (5 µM) was applied to MDA-MB-231, MDA-MB-468, SKBR-3, MCF7 cells for 1 week. The values are represented as survival fraction, the percentage where the PBS-treated group were regarded as 100%. Data are mean ± SD (n = 5). ****P* < 0.001, *vs* PBS-treated group.


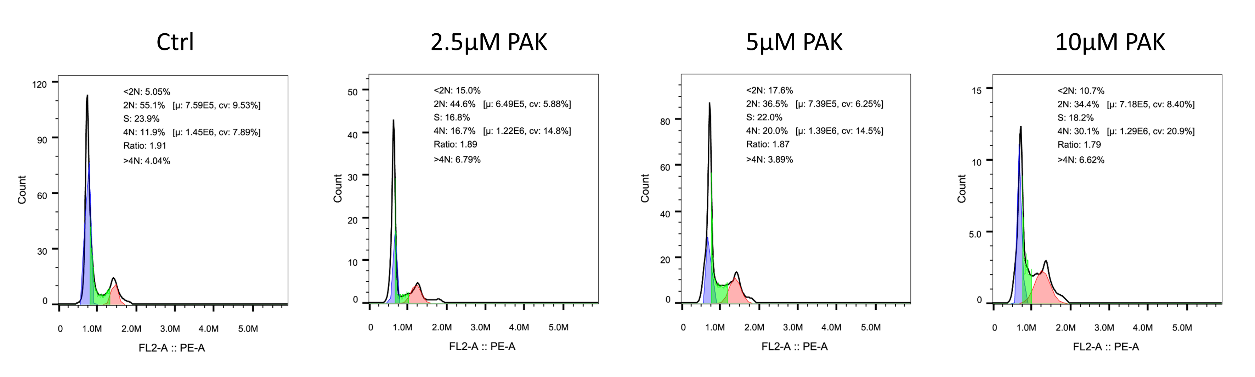


**Figure S3.** After 24-hour treatment, the effect of PAK on MDA-MB-231 cell-cycle distribution was measured.


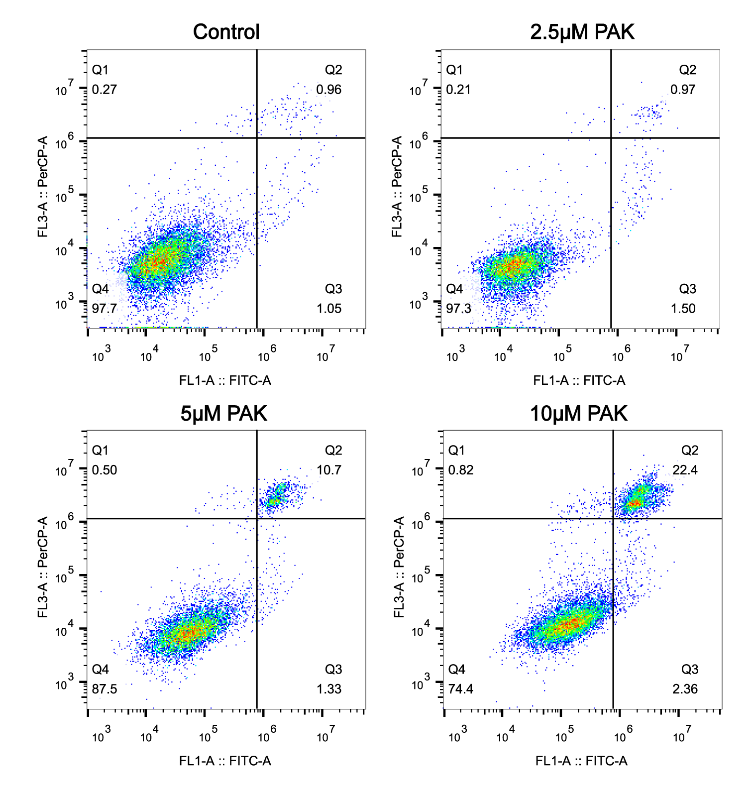


**Figure S4.** Relative cell densities of viable and apoptotic Annexin V positive cells were compared to untreated control cells after 24 hours of treatment**.**


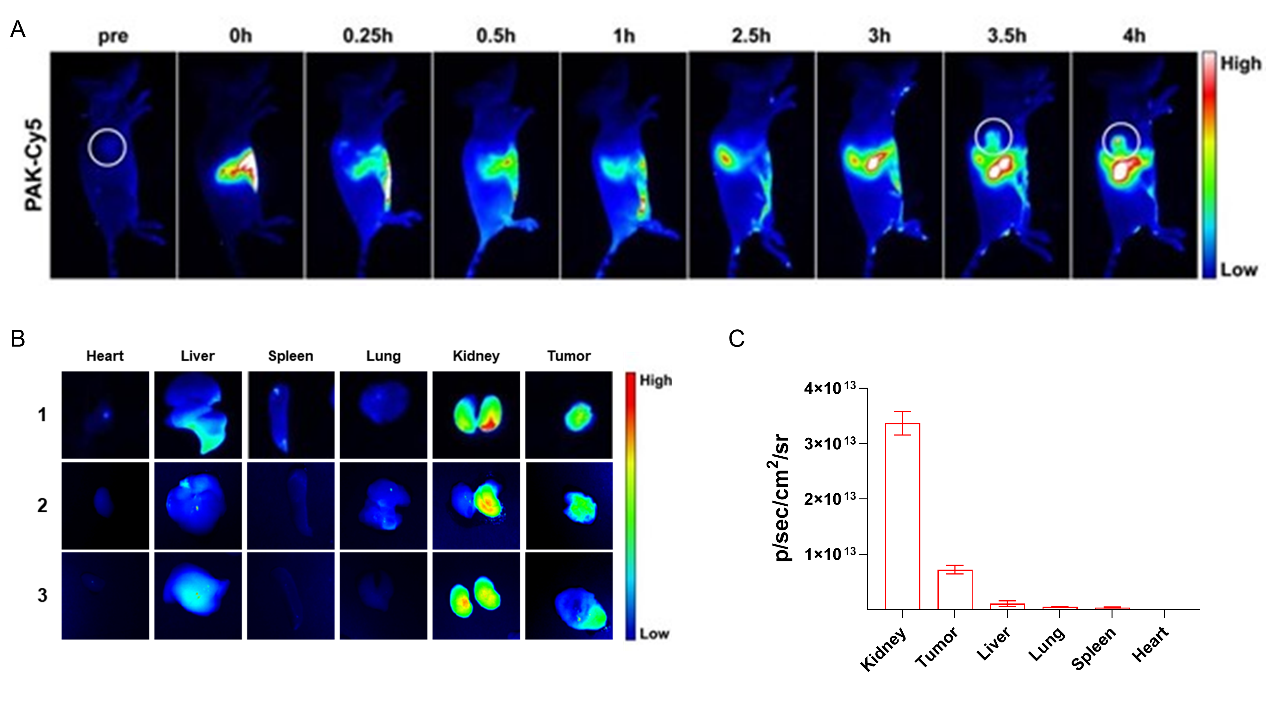


**Figure S5. Study on the targeted tumor ability of PAK *in vivo*. (A)** *In vivo* imaging results at different time points in mice. **(B-C)** Imaging and quantitative fluorescence results of dissected heart, liver, spleen, lung, kidney, and tumor. Data are mean ± SD (n = 3).

**
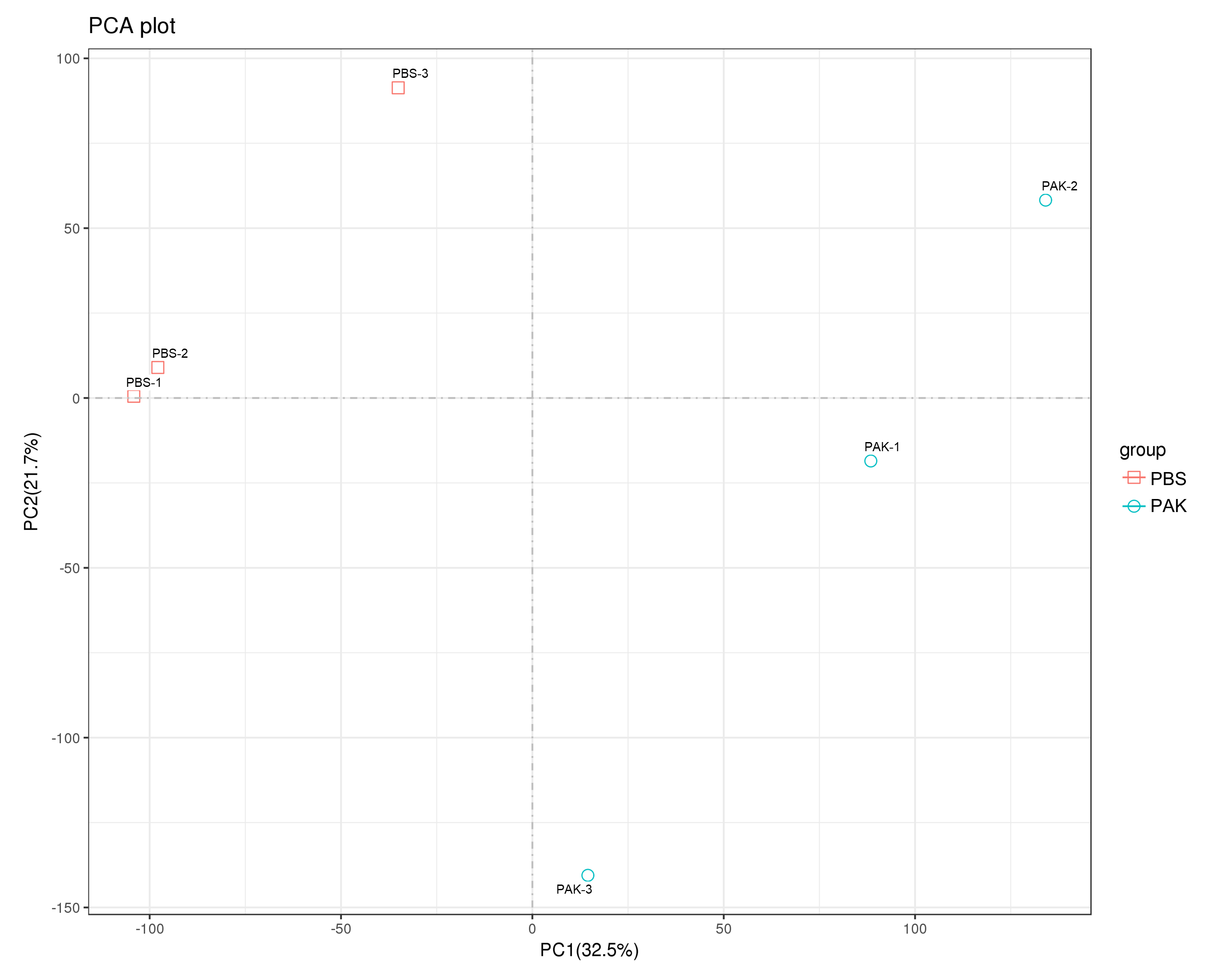
**

**Figure S6. Principal component analysis.** The position of each point represents the value of the sample on each principal component.

**
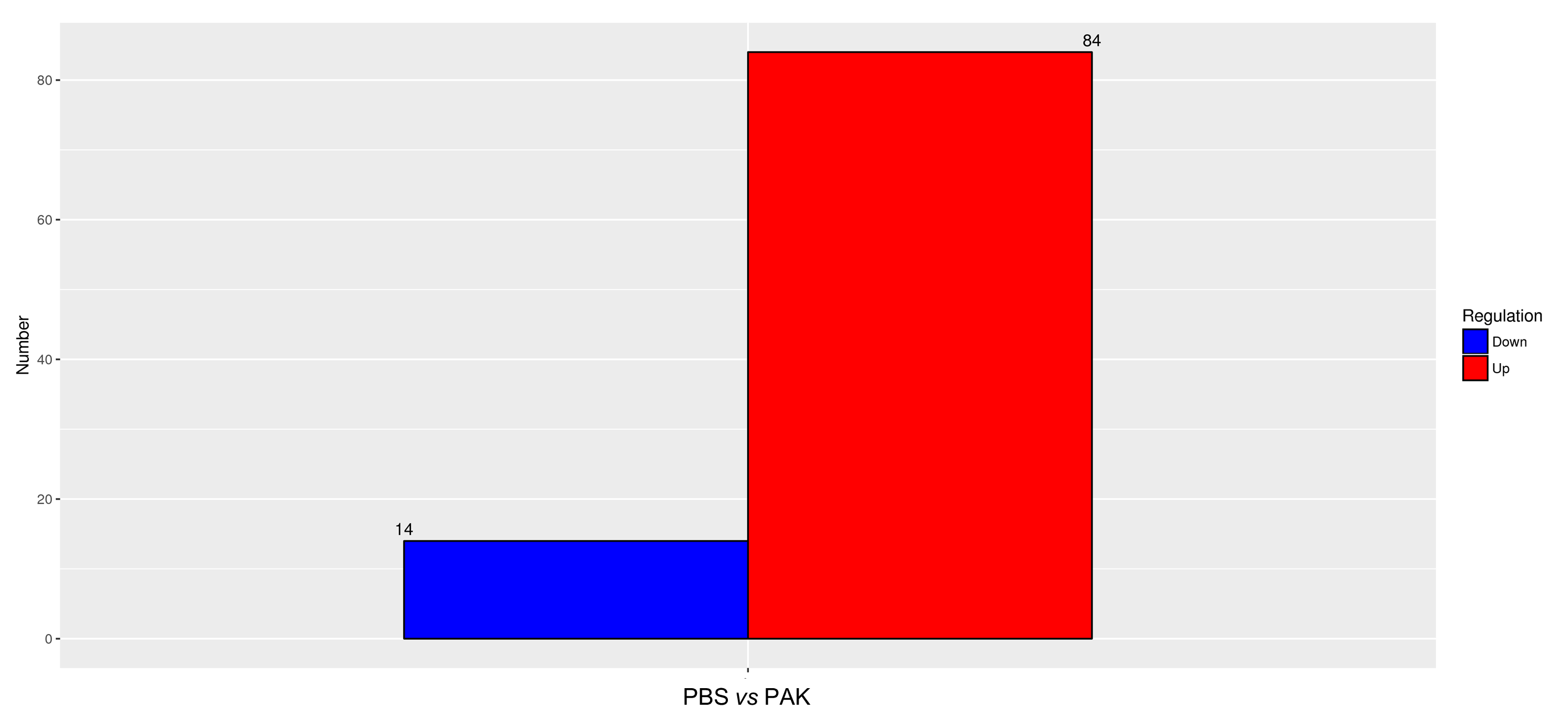
**

**Figure S7.** Differential gene expression between samples was compared to assess upregulation and downregulation.


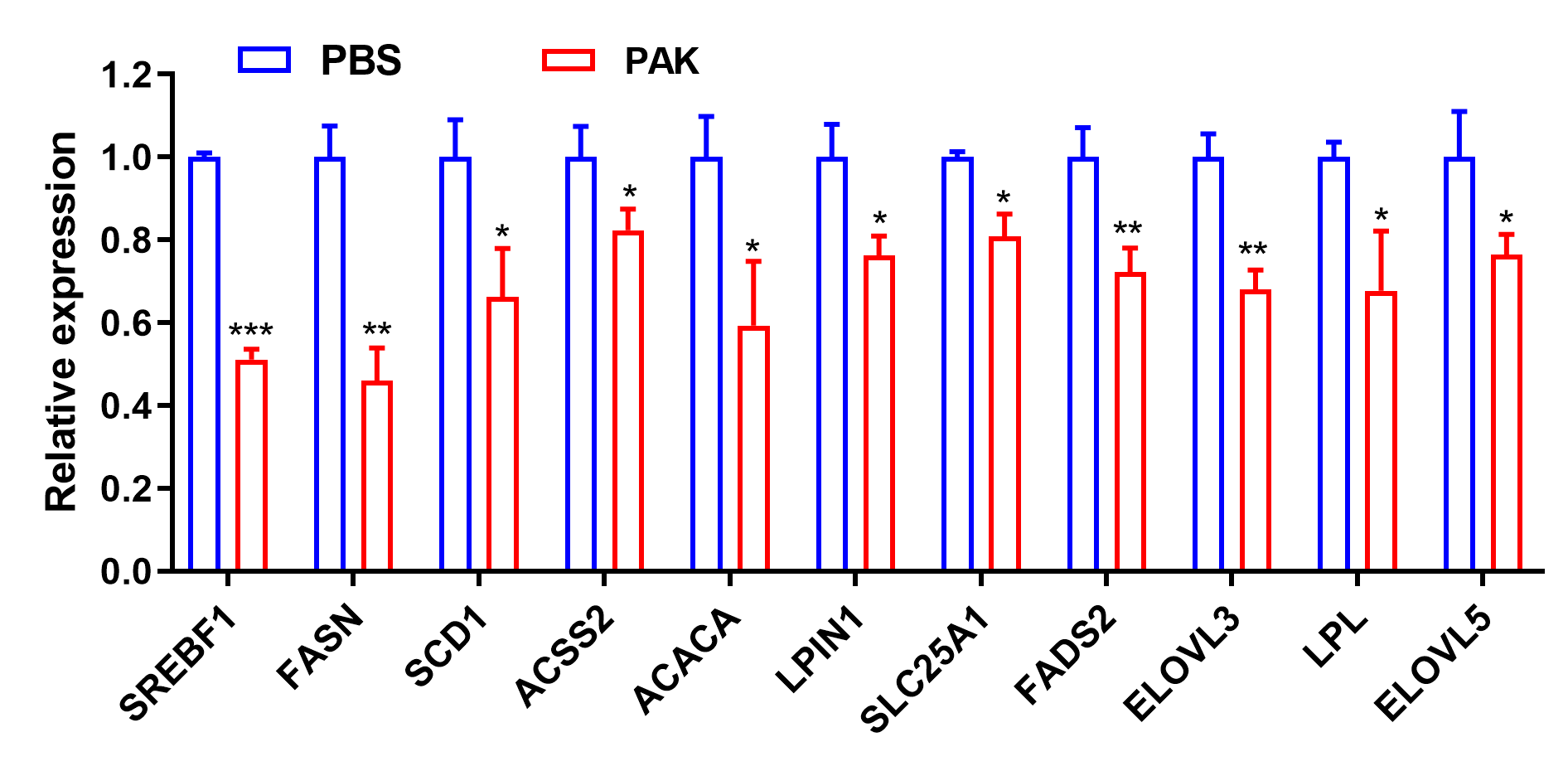


**Figure S8.** Relative expression levels in differential expressed genes related to fatty acid synthesis were calculated, where the control group were regarded as 100%. Data are mean ± SD (n = 3). **P* < 0.05; ***P* < 0.01; ****P* < 0.001.


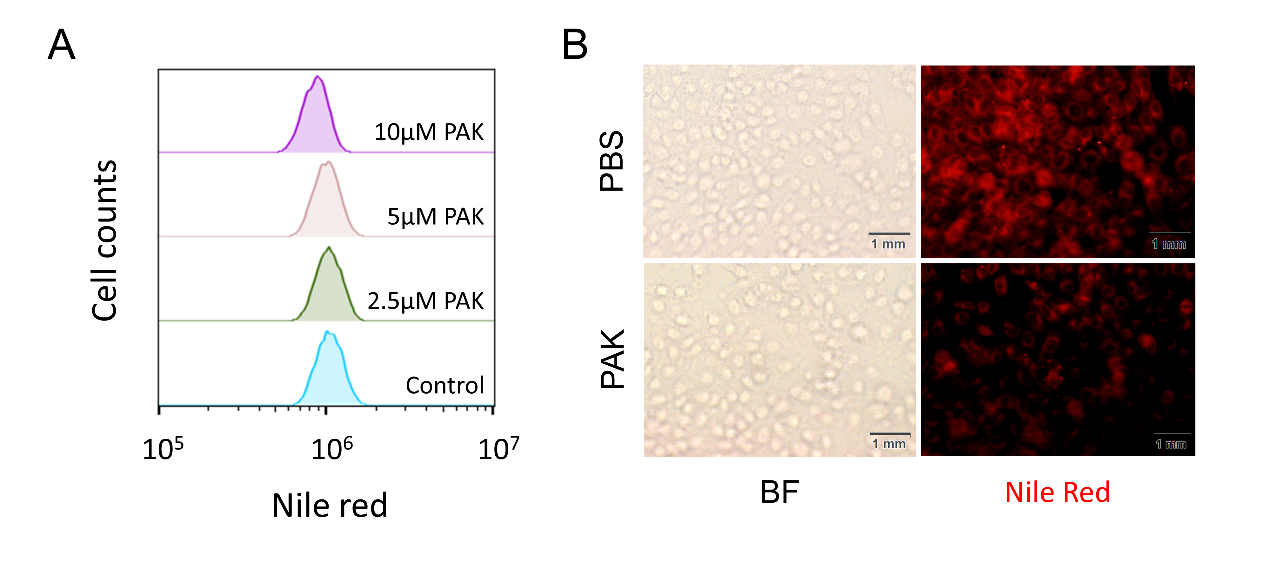


**Figure S9.** Nile red fluorescence intensity distribution in MDA-MB-231 cells treated with PBS or PAK (5 µM) for 24 hours were measured.


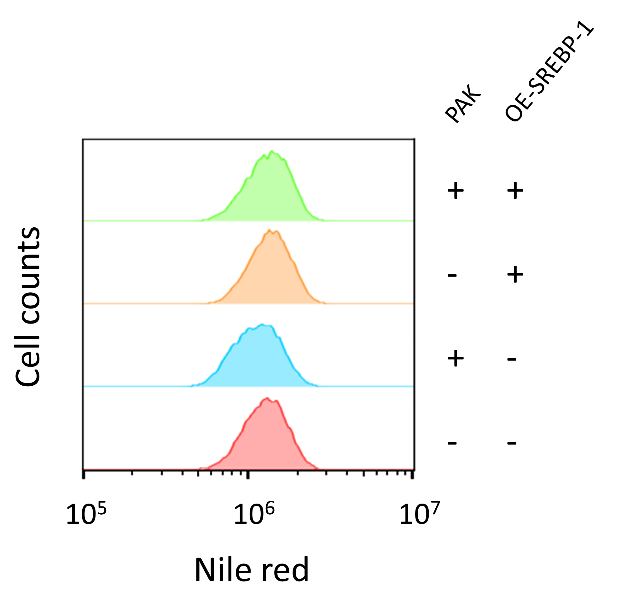


**Figure S10.** Nile Red fluorescence intensity distribution in MDA-MB-231 cells treated with PAK (5 µM) or/and OE-SREBP-1 were measured.

1. **Supplement Images**

Figure 1H

| **Name** | **Blot images** |
| --- | --- |
| N-cadherin | 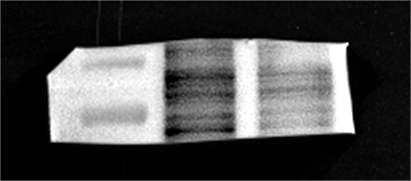 |
| E-cadherin | 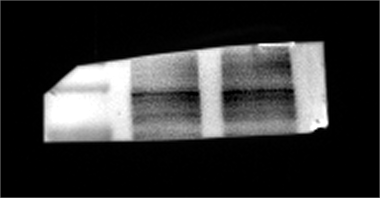 |
| Vimentin | 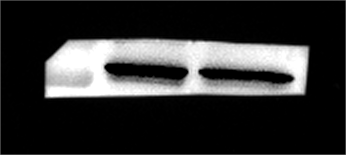 |
| β-actin | 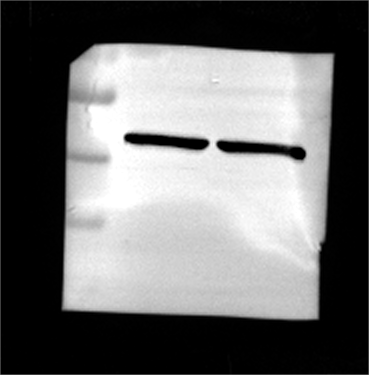 |

Figure 3E

| **Name** | **Blot images** |
| --- | --- |
| SREBP-1(P) | 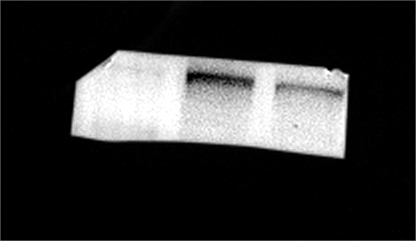 |
| SREBP-1(N) | 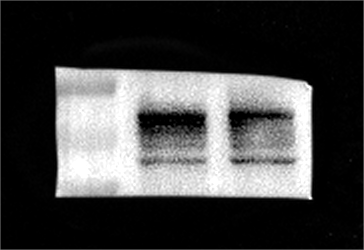 |
| FASN | 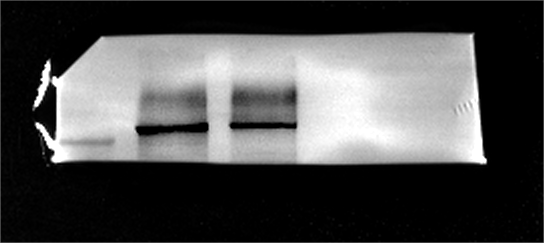 |
| SCD1 | 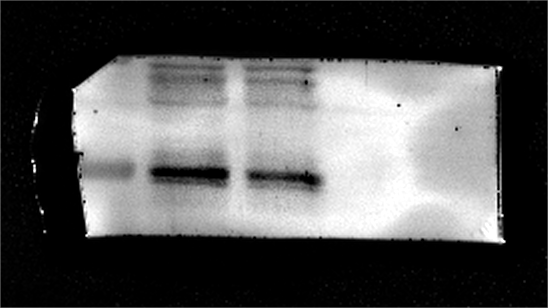 |
| β-actin | 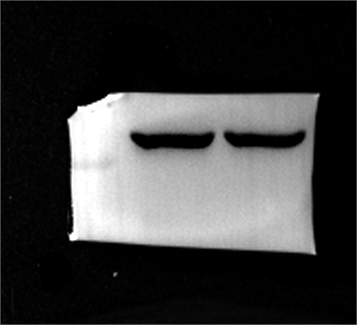 |

Figure 5A

| **Name** | **Blot images** |
| --- | --- |
| SREBP-1(P) | 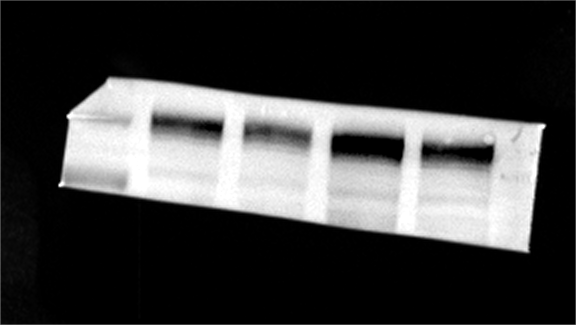 |
| SREBP-1(N) | 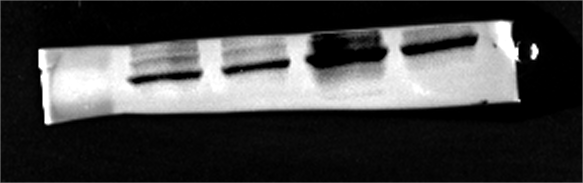 |
| FASN | 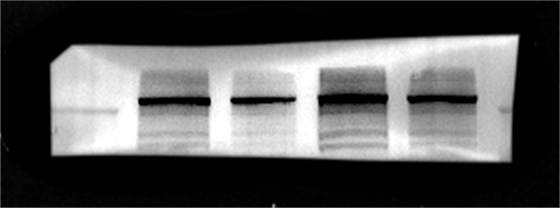 |
| SCD1 | 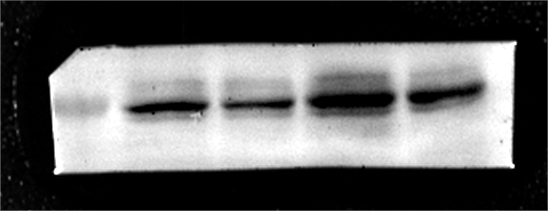 |
| β-actin | 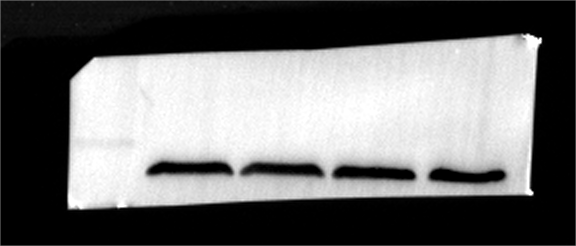 |
